# Orofacial abnormalities in mucopolysaccharidosis and mucolipidosis type II and III: A systematic review

**DOI:** 10.1002/jmd2.12331

**Published:** 2022-09-21

**Authors:** Chiel J. de Bode, Emma J. Dogterom, Antoinette V. J. Rozeboom, Janneke J. Langendonk, Eppo B. Wolvius, Ans T. van der Ploeg, Esmée Oussoren, Margreet A. E. M. Wagenmakers

**Affiliations:** ^1^ Department of Oral & Maxillofacial Surgery Erasmus MC University Medical Center Rotterdam The Netherlands; ^2^ Department of Pediatrics, Center for Lysosomal and Metabolic Diseases, Erasmus MC University Medical Center Rotterdam Rotterdam The Netherlands; ^3^ Department of Internal Medicine, Center for Lysosomal and Metabolic Diseases, Erasmus MC University Medical Center Rotterdam Rotterdam The Netherlands

**Keywords:** mucolipidosis type II, mucolipidosis type III, mucopolysaccharidosis, orofacial abnormalities

## Abstract

Mucopolysaccharidoses (MPSs) and mucolipidosis II and III (ML II and III) often manifest with orofacial (progressive) abnormalities, which may have a major impact on quality of life. However, because these patients have multiple somatic health issues, orofacial problems are easily overlooked in clinical practice and available literature on this topic solely consists of case reports, small case series, and small cohort studies. The aim of this systematic review was to gain more insight in the nature and extent of orofacial abnormalities in MPS, ML II, and III. A systematic review of all previously published articles addressing orofacial abnormalities in MPS, ML II, and III was performed. Both clinical studies and case reports were included. Outcome was the described orofacial abnormalities, subdivided into abnormalities of the face, maxilla, mandible, soft tissues, teeth, and occlusion. The search resulted in 57 articles, describing orofacial features in 340 patients. Orofacial abnormalities were present in all subtypes of MPS, ML II, and III, and consisted of thickened lips, a hypoplastic midface, a high‐arched palate, hypoplastic condyles, coronoid hyperplasia, macroglossia, gingival hyperplasia, thick dental follicles, dentigerous cysts, misshapen teeth, enamel defects, and open bite. Orofacial abnormalities are present in all subtypes of MPS, ML II, and III. As orofacial abnormalities may cause complaints, evaluation of orofacial health should be part of routine clinical care.

**Synopsis**

As orofacial abnormalities are present in all types of mucopolysaccharidosis, mucolipidosis II, and III, evaluation of orofacial health should be part of routine clinical care in these patients.

## 1 INTRODUCTION

For physicians who treat patients with mucopolysaccharidosis (MPS) and mucolipidosis types II and III (ML II and III), it is well known that orofacial abnormalities may be a relevant clinical issue in certain patients. Because these patients have multiple somatic health issues, orofacial problems are easily overlooked in clinical practice and no guidelines on treatment and follow‐up exist.

MPS, ML II, and III are rare lysosomal storage disorders. In MPS, ML II, and III, the accumulation of glycosaminoglycans (GAGs) in lysosomes in connective tissue, cartilage, bones, ligaments, and other tissues results in cellular dysfunction, finally leading to tissue damage and organ dysfunction. This leads to multisystemic changes, ranging from skeletal abnormalities (so called dysostosis multiplex), cardiorespiratory disease and progressive neurocognitive dysfunction. The combination and severity of symptoms in an individual patient depend on the type of MPS, ML II, or III and whether residual enzyme activity is present.^1^, ^2^, ^3^

There are 11 different types of MPS, all caused by deficiencies of different GAG‐degrading enzymes. Depending on which enzyme is deficient, different GAGs such as heparan, dermatan, chondroitin, and keratan sulfate, accumulate in lysosomes.^4^

ML II and III are caused by deficiency of the same enzyme *N‐*acetylglucosamine‐1‐phosphotransferase. This enzyme phosphorylates mannose to mannose‐6‐phosphate, which is responsible for the transport of more than 70 lysosomal hydrolases. The absence of these enzymes in the lysosomes results in nondegraded macromolecules accumulation, such as GAGs.^5^, ^6^, ^7^, ^8^, ^9^, ^10^ ML II is characterized by total loss of enzyme activity with a severe phenotype, whereas ML III has residual enzyme activity, resulting in a milder phenotype.^11^

MPS, ML II, and III often manifest with orofacial abnormalities. GAG accumulation in soft tissues, cartilage, and bones and secondary cellular responses to accumulated GAGs may lead to abnormalities in orofacial soft tissues, orofacial bones, and teeth. The majority of orofacial bones are developed by intramembranous ossification, but the mandible's condyle and symphysis are formed by endochondral ossification.^12^ Both intramembranous and endochondral ossification are affected in MPS, ML II, and III. As tooth development shares similarities with bone development, alterations would be expected in teeth as well.^3^, ^12^, ^13^, ^14^, ^15^

Available literature on orofacial abnormalities in MPS, ML II, and III consists of case reports, case series, and cohort studies, with limited patient numbers included. It is unclear if and to what extent orofacial abnormalities occur in all subtypes of MPS, ML II, and III and if the type of abnormalities differ between different subtypes, and what percentage of patients is affected.

Other disorders presenting with orofacial abnormalities have clearly demonstrated that orofacial abnormalities can have a large impact on quality of life, with functional and psychological consequences.^16^, ^17^, ^18^, ^19^, ^20^, ^21^, ^22^ To better understand orofacial abnormalities in patients with MPS, ML II, and III, knowledge about the prevalence and types of orofacial abnormalities in the different subtypes of MPS, ML II, and III is essential. In order to gain more insight in the nature and extent of orofacial abnormalities in MPS, ML II, and III we performed a systematic review of the literature.

## 2 METHODS

A systematic literature search was conducted of the following electronic databases on 30 April 2020: EMBASE, Medline via Ovid, Web of Science Core Collection, Cochrane Central Registry of Trials, and Google Scholar. The search strategy was structured using the keywords “mucopolysaccharidosis,” “mucolipidosis type 2,” “mucolipidosis type 3,” “face malformation,” and “mouth disease.” The full search strategy is shown in Supplemental material S1. Two reviewers (Chiel J. de Bode and Emma J. Dogterom) independently assessed the titles and abstracts, and if it was unclear from title and abstract the whole article, to decide whether articles were suitable for inclusion. Any disagreements were discussed between the two reviewers. Publications were included based on clinical studies and case reports published in peer reviewed journals, with orofacial feature descriptions in patients with proven MPS, ML II, or III in human patients, containing original data of one or multiple patients, and written in English. Reviews and conference abstracts were excluded, as well as articles describing an unclear diagnosis. Double registration of cases was likely eliminated by comparing authors, hospitals, country of publication, and patient characteristics.

### 2.1 Data extraction

Abnormal phenotype and orofacial characteristics were extracted from included articles and entered into a database. Orofacial characteristics were defined as characteristics of the face, maxilla, mandible, soft tissues, teeth, and occlusion.

### 2.2 Statistical analyses

The data were analyzed using the statistical software packages SPSS statistics version 25. The total number of patients and their different phenotypes and orofacial abnormalities were calculated. All results were descriptive, presented as number of patients in whom orofacial abnormalities were reported.

## 3 RESULTS

**FIGURE 1:**
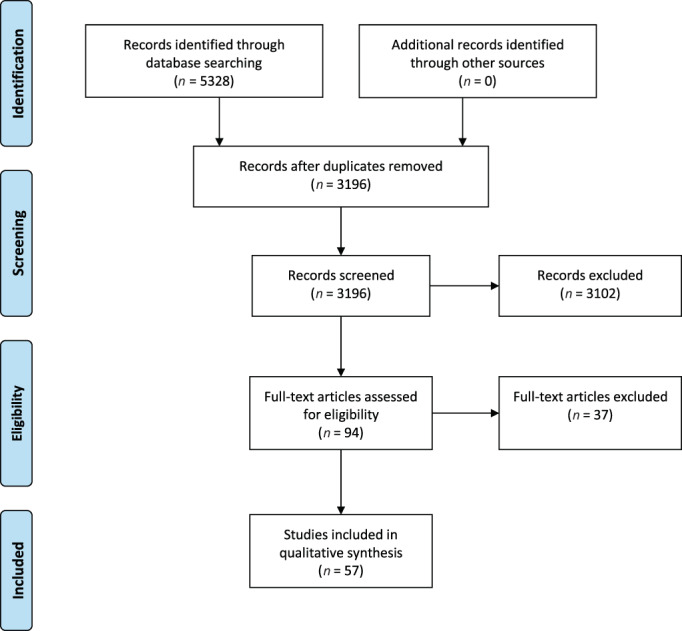
Study selection flow diagram

**FIGURE 2:**
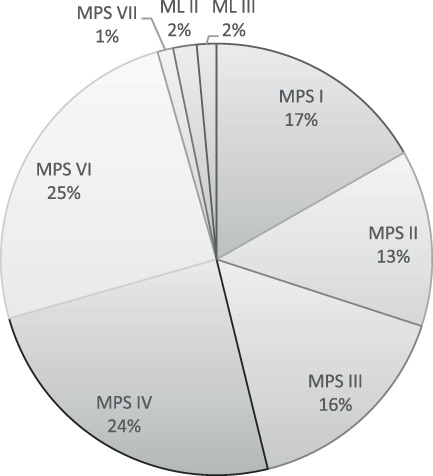
Distribution of mucopolysaccharidosis (MPS) and mucolipidosis (ML) types of pateints (*n* = 340) included in this review

A total of 5328 records were identified through database searching, after checking on duplicates 3196 remained. A total of 94 publications were included based on title and abstract, of which the full text was screened. In total, 37 of these 94 articles were excluded for three different reasons; (1) no clinical orofacial patient characteristics (*n* = 17), (2) patients without a certain diagnosis of MPS, ML II, or III (*n* = 9), and (3) lack of original data (*n* = 11). This resulted in 57 articles that were included for data extraction (Figure 1). These 57 articles contained orofacial features of 340 patients with MPS, ML II, or III. The articles included were published between 1972 and 2020. A total of 32 articles were single case reports, 15 case series (number of included cases = 46) and 10 clinical studies (number of included cases = 262). A list of the references of the publications included in our database is shown in Supplemental Material S2. The full articles of all included papers were available for our analysis. In Figure 2, the distribution of MPS, ML II, and III patients in our database are described. In total, 17% of the patients were diagnosed with MPS I (*n* = 57), 13.2% MPS II (*n* = 45), 16.2% MPS III (*n* = 55), 24.4% MPS IV (*n* = 83), 25.0% MPS VI (*n* = 85), 1.2% MPS VII (*n* = 4), 1.8% ML II (*n* = 6), and 1.5% ML III (*n* = 5).

### 3.1 Face

All subtypes of MPS and ML II manifested with at least one facial abnormality. Abnormal features that were mentioned most frequently were a prominent forehead (*n* = 8/340), hypertelorism (*n* = 14/340), a flattened nasal bridge (*n* = 24/340), thickened lips (*n* = 35/340), a hypoplastic midface (*n* = 26/340), a convex profile of the face (*n* = 29/340), and a prominent lower third of the face (*n* = 21/340). A full overview of all facial characteristics is shown in Supplemental Material S3. In all patients with ML III (*n* = 5) none of these features were reported.

### 3.2 Maxilla

In Table 1, an overview is given of the number of MPS and ML patients in whom orofacial features were described. A high‐arched palate was described in MPS I, II, III, VI, and ML II patients (*n* = 13/340). Various other anomalies in palatal anatomy, including horizontal and/or vertical grooves and U, V, flat, or broad‐shaped palatal vaults were described in only one of the studies. This study investigated a specific subgroup of patients: patients with MPS I after hematopoietic stem cell transplantation.^23^ A narrow maxilla was described in nine MPS VI patients (*n* = 9/85).

**TABLE 1:** Reported orofacial abnormalities in MPS, ML II, and III

	MPS I (*n* = 57)	MPS II (*n* = 45)	MPS III (*n* = 55)	MPS IV (*n* = 83)	MPS VI (*n* = 85)	MPS VII (*n* = 4)	ML II (*n* = 6)	ML III (*n* = 5)
**Palate and maxilla**								
High‐arched palate	1	2	1	—	8	—	1	—
Narrow Maxilla	—	—	—	—	9	—	—	—
Mandible								
Condylar defect	21	11	—	16	37	4	—	2
Flattened condyle head	9	2	—	12	10	—	—	1
Hypoplastic condyle	5	6	—	1	24	3	—	1
Limited mouth opening	11	13	13	11	24	—	—	2
Short ramus	13	5	—	3	9	1	—	—
Coronoid process defect	1	7	—	3	6	1	—	—
Thin inferior cortex	1	1	—	—	10	—	—	—
**Soft tissues**								
Macroglossia	16	14	1	5	15	1	3	—
Gingiva hyperplasia	7	7	2	2	15	—	5	—
Thick dental follicle	4	4	—	—	39	3	—	2
Dentigerous cysts	1	2	—	—	14	—	—	1
**Teeth**								
Diastema	24	15	1	13	21	1	—	—
Misshapen	30	6	1	11	18	3	—	—
Enamel defect	10	1	1	51	2	—	1	—
Impacted/unerupted teeth	1	9	—	4	24	1	1	2
Delayed eruption/prolonged retention	12	8	1	2	15	—	1	1
Malposition	—	1	—	—	24	3	—	—
Absence	18	1	—	1	1	—	2	2
Ectopic teeth	7	4	—	—	2	—	—	1
Crowding	5	2	1	2	1	—	—	—
Supernumerary	1	1	—	1	7	—	—	—
Rotation/giroversion	1	—	—	—		—	—	1
Root malformation	10	2	—	—	10	1	—	—
**Misshapen teeth**								
Taurodontism	7	1	—	2	13	3	—	—
Peg‐shaped teeth	3	2	1	1	2	—	—	—
Pointed molar cusps	5	—	—	14	—	—	—	—
Microdontia	16	—	—	—	1	—	—	—
Pointed cuspids	10	2	—	2	—	—	—	—
Spade‐shaped incisors	—	—	—	10	—	—	—	—
**Occlusion**								
Anterior open bite	15	12	2	12	30	3	2	1
Cross bite	4	7	1	8	6	1	—	—
Class I malocclusion	11	1	—	1	—	—	—	—
Class II malocclusion	8	—	—	—	—	—	—	1
Class III malocclusion	6	2	—	—	2	—	—	—

*Note*: Data are presented as number of patients reported in literature with different types of orofacial abnormalities. As data have been extracted from case reports/series or small cohort studies with different outcome measures and without thorough description of the phenotype of included patients, no data on prevalence can be calculated.

Abbreviations: ML, mucolipidosis; MPS, mucopolysaccharidosis.

### 3.3 Mandible

Mandible abnormalities were described in both MPS and ML patients. Condylar defects were observed in MPS I, II, IV, VI, and VII and in ML III patients (*n* = 91/340). Condylar defects were further specified to hypoplastic condyles (*n* = 40/91) and/or flattening of the condyle head (*n* = 34/91). A limited mouth opening was described in 21.8% of patients (*n* = 74/340), this was related to a condylar defect in 11 patients. A short ramus bone (*n* = 31/340), coronoid process defects (*n* = 18/340), and thin inferior cortex (*n* = 12/340) were only described in MPS patients.

### 3.4 Soft tissues

**FIGURE 3:**
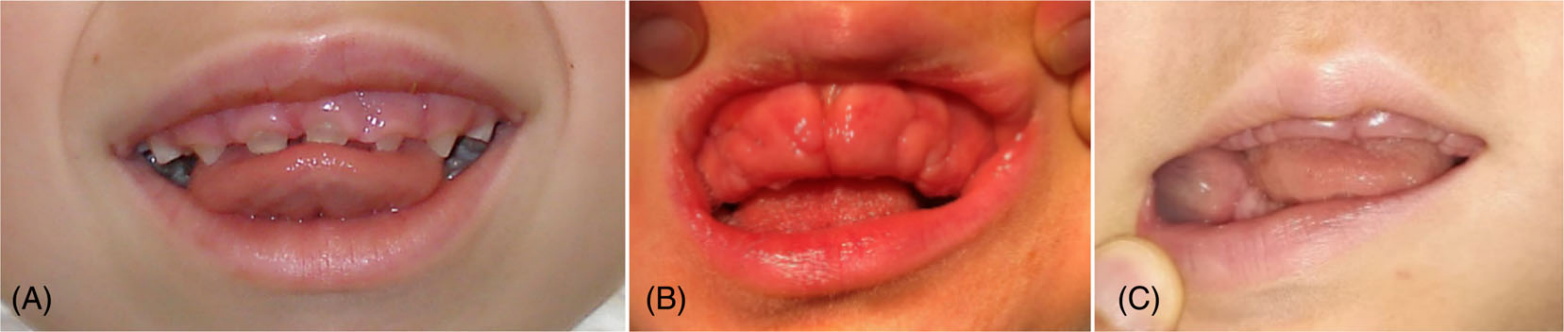
(A) Enamel defects and peg‐shaped teeth in a 6‐year‐old mucopolysaccharidosis I patient. (B) Gingiva hyperplasia in a 2‐year‐old mucolipidosis (ML) II patient. (C) Dentigerous cyst in a 2‐year‐old ML II patient.

Macroglossia was described in all MPS types and in ML II (*n* = 55/340). Gingiva hyperplasia was mentioned in all types (*n* = 38/340; Figure 3). Thick dental follicles (*n* = 52/340) and dentigerous cysts (*n* = 18/340; Figure 3) were mostly described in MPS VI patients.

### 3.5 Teeth

Described teeth abnormalities were diastema (*n* = 75/340), misshapen teeth (*n* = 69/340), enamel defects (*n* = 66/340) (Figure 3), impacted teeth (*n* = 42/340), delayed tooth eruption (*n* = 40/340), malposition of teeth (*n* = 28/340), absence of teeth (*n* = 25/340), ectopic teeth (*n* = 14/340), crowding (*n* = 11/340), supernumerary teeth (*n* = 10/340), and rotation of teeth (*n* = 4/340).

Misshapen teeth include molars and could be specified as taurodontism (*n* = 26/69), peg‐shaped teeth (*n* = 19/69) (Figure 3), pointed molar cusps (*n* = 19/69), microdontia (*n* = 17/69), pointed cuspids (*n* = 14/69), and spade‐shaped incisors (*n* = 10/69). Spade‐shaped incisors were only described in MPS IV patients.

Root malformations were only described in MPS patients (*n* = 23/340), these were root dilacerations, short and blunted tooth roots, and long tooth roots.

### 3.6 Occlusion

Anterior open bite was reported in all types of MPS, ML II, and III patients (*n* = 77/340) and cross bite in all MPS types (*n* = 27/340). Cross bite was rarely specified, posterior cross bite was reported the most (*n* = 13/27). Classes I (*n* = 13), II (*n* = 9), and III (*n* = 10) malocclusions were described in in both MPS and ML patients.

## 4 DISCUSSION

This systematic review gives an overview of the extent and nature of orofacial abnormalities, described in MPS, ML II, and III patients. Despite the rarity of these diseases, multiple articles describe orofacial abnormalities in these patients.

The results from this systematic review demonstrate that orofacial abnormalities are present in all types of MPS, ML II, and III. However, most information is based on case series or case reports that did not describe orofacial abnormalities in a systematic manner. Ten clinical studies were included, but these papers missed systematic information on the severity of the phenotypes and used different outcome measures.^23^, ^24^, ^25^, ^26^, ^27^, ^28^, ^29^, ^30^, ^31^, ^32^ Reliable comparison of the prevalence, severity and clinical consequences of orofacial abnormalities was impossible. We therefore presented the orofacial abnormalities in a descriptive manner.

Facial abnormalities such as a prominent forehead or a hypoplastic midface in MPS can be explained by aberrant growth of both intramembranous and endochondral bone.^3^ Two studies presented results of cephalometric characteristics of MPS I (*n* = 1), MPS IV (*n* = 8), and MPS VI (*n* = 9) patients. These studies showed that MPS patients can manifest with a tendency towards vertical growth of their face resulting in a dolichocephalic facial pattern (a relatively long face).^33^, ^34^ A high‐arched palate and a narrow maxilla have been found as well. Turra et al.,^35^ found a high‐arched palate in 71.2% (*n* = 47) of MPS patients in a cross‐sectional study, but this study was not included in our database, because the MPS type was not specified. Case descriptions contained little information about maxillary characteristics of MPS and ML patients. There were descriptions of significant maxillary hypoplasia, and maxillary narrowing in excluded articles, data not shown.^13^, ^36^ Concerns with facial abnormalities are potential breathing problems. Maxillary hypoplasia seen in MPS I patients was suggested to contribute to obstructive sleep apnea syndrome (OSAS).^36^ Furthermore, a narrowed oropharynx and nasopharynx was mentioned in two MPS VI patients. Aberrant growth of the face, together with a decreased nasopharyngeal space may lead to breathing problems.^33^ Besides maxillary abnormalities, mandible abnormalities correlated to respiratory difficulties have been found. A high prevalence of OSAS (60%–75%) has been found by Koehne et al.^36^ in MPS I and VI patients, which was correlated to mandibular micrognathia. Also macroglossia can contribute to breathing disorders.^37^, ^38^

Condyle defects can also develop from GAG accumulation in the temporomandibular joint (TMJ), resulting in flattening and erosion of the condylar heads. These defects can result in headache, ear pain, pain on palpation, clicking sounds during mouth opening, and open bite.^25^, ^29^, ^39^ An open bite, observed in these patients, could be related to an enlarged tongue, as well as to condylar hypoplasia.^25^, ^29^, ^40^ The latter is confirmed by observations of De Almeida‐Barros et al.^25^ and Kantaputra et al.,^29^ who observed a relation between condylar hypoplasia and open bite. The changes of the condyle led to decreased mobility of the TMJ, resulting in an open bite, which can lead to severe chewing problems and choking.^25^, ^41^, ^42^ Coronoid hyperplasia can lead to limited mouth opening.^43^ A recently published case report described a 14 year‐old MPS VI patient with an interincisal distance of 8 mm with coronoid hyperplasia. This intervened with eating, speech, oral hygiene, and intubation. The given treatments, a bilateral coronoidectomy and physiotherapy, were successful and increased the mouth opening to 45 mm.^43^

Soft tissue abnormalities were present in all MPS types and in ML II and III. Macroglossia can lead to breathing and eating problems, slowed speech development and malocclusions like diastema and labial inclination of anterior teeth.^37^, ^38^

The gingival hyperplasia observed in MPS and ML II patients is associated with delayed eruption of teeth, which is described in MPS, ML II, and III patients as well.^44^ Other soft tissue abnormalities in MPS and ML patients are thick dental follicles and dentigerous cysts. Impaction and delayed eruption of teeth is found to be secondary to thickened cyst‐like follicles as well.^25^, ^45^ These follicles present as radiolucent areas with smooth, clearly defined margins, are limited to the crowns and consist of an increased amount of GAGs.^39^, ^40^, ^46^, ^47^ The appropriate treatment for this lesion is surgical exposure of the impacted teeth.^47^ Dentigerous cysts are larger, cause bone destruction, and press teeth out of position. The treatment for dentigerous cysts can be either marsupialization if the involved teeth should be preserved or enucleation with the involved teeth.^47^

When compared with healthy controls, MPS patients manifest significantly more often with misshapen teeth and enamel hypoplasia.^13^, ^48^ Hypoplastic enamel consists of thin enamel, but with normal radiodensity, the enamel defects can manifest in both primary and permanent dentition.^28^, ^30^, ^41^, ^49^ Enamel hypoplasia is typically a feature of MPS IV‐A, but has also been described in other types of MPSs and in ML II.^25^, ^32^, ^50^ Pointed molar cusps are mostly described in MPS IV patients, which is most likely caused by the thin enamel in these patients.^41^ Furthermore, MPS IV patients have been described as having an increased oral health need due to increased caries, related to the enamel defects.^28^

Class I, II, and III malocclusions have been described. The changes in occlusal relationships could be caused by the defects in orofacial bone development.^25^, ^29^, ^34^, ^40^, ^51^ A finding within these malocclusions is flaring of the anterior teeth.^23^, ^30^, ^37^, ^52^ A cause of this labial inclination of the anterior teeth can be a tongue thrust habit.^37^

A major limitation of this review is the low quality of evidence available on orofacial abnormalities in MPS, ML II, and III, since the data used in this study are mainly based on case reports or series or small cohort studies with different outcome measures and without thorough description of the phenotype of included patients. Therefore, the results likely represent a gross underestimation of the actual prevalence of orofacial abnormalities in MPS, ML II, and III patients.

In conclusion, this review demonstrates that orofacial abnormalities are common in patients with MPS, ML II, and III and present in all subtypes. Information of actual clinical relevance is missing, this can be either somatically, functionally, or psychologically. The differences between subtypes and phenotypes are still unclear. At present it is impossible to generate an evidence based follow‐up protocol for orofacial care, with the aim to improve clinical care and quality of life of the patients. Based on studies in other diseases that present with orofacial abnormalities, we are convinced that it is very likely that such a protocol may be of great added value. A systematical cohort study, with systematically and objectively described abnormalities and their functional and somatic consequences and the impact on quality of life, is the next step.

## CONFLICT OF INTEREST

Chiel J. de Bode, Emma J. Dogterom, Antoinette V. J. Rozeboom, Janneke J. Langendonk, Eppo B. Wolvius, Ans T. van der Ploeg, Esmée Oussoren, and Margreet A. E. M. Wagenmakers declare that they have no conflict of interest.

## ETHICS STATEMENT

No ethics approval was obtained since the work consisted of a systematic literature search, for which no ethical approval is necessary in the institutions/countries participating in the study.

## INFORMED CONSENT AND ANIMAL RIGHTS

This article does not contain any studies with human or animal subjects performed by any of the authors.

## Supporting information


**Appendix S1**. Supporting Information

## Data Availability

Data supporting the results can be found in the supplementary materials.
