# Supplementary material for: Orofacial abnormalities in mucopolysaccharidosis and mucolipidosis type II and III: A systematic review
Source: JIMD Rep. 2022 Sep 21;63(6):621–9. doi: 10.1002/jmd2.12331 (PMC9626671; doi:10.1002/jmd2.12331)
Supplement: Supplementary file 1 — Appendix S1. Supporting Information [file JMD2-63-621-s001.docx]

**S1**

**Supplemental 1** Search strategies April 2020

| *Database* | *References* | *After de-duplication* |
| --- | --- | --- |
| Embase.com | 1836 | 1822 |
| Medline (Ovid) | 1724 | 631 |
| Web of Science Core Collection | 1416 | 526 |
| Cochrane Central Registry of Trials | 152 | 88 |
| Google Scholar (top 200 relevance rank) | 200 | 134 |
| **Total** | **5328** | **3201** |

**Embase.com**

('face malformation'/de OR 'mouth disease'/exp OR 'tooth disease'/exp OR 'mouth malformation'/exp OR 'jaw malformation'/de OR 'mandible hypoplasia'/de OR 'maxilla hypoplasia'/de OR 'micrognathia'/de OR 'prognathia'/de OR 'retrognathia'/de OR 'maxilla'/de OR 'dental surgeon'/de OR (maxill* OR macroglossia* OR macrostomia* OR microstomia* OR mouth* OR oral* OR lip* OR jaw* OR palate* OR tooth* OR teeth* OR dental* OR gingiva* OR tongue* OR enamel* OR temporomandibular-joint* OR stomatognathic-complex* OR caries* OR ((odontogenic* OR eruption* OR dentigerous*) NEAR/3 (cyst*)) OR ((mandible**) NEAR/3 hypoplasia*) OR micrognathia* OR prognathia* OR retrognathia*):ab,ti,kw) AND ('mucopolysaccharidosis'/de OR 'Hunter syndrome'/de OR 'Hurler syndrome'/de OR 'Maroteaux Lamy syndrome'/de OR 'Morquio syndrome'/de OR 'mucopolysaccharidosis type 7'/de OR 'Sanfilippo syndrome'/de OR 'Scheie syndrome'/de OR 'mucolipidosis'/exp OR (mucolipidos* OR mucopolysaccharidos* OR MPS OR Scheie* OR (Hunter* NEAR/3 (syndrome* OR disease*)) OR Hurler* OR Maroteaux-Lamy* OR Morquio* OR Sanfilippo OR Sly* OR natowicz* OR ((acetylglucosamin*) NEAR/6 (phosphotransferas*) NEAR/6 (syndrome* OR deficien*)) OR i-cell-disease* OR Leroy-disease* OR glcnac*-ptase*-deficien* OR GNPTAB OR GNPTG OR pseudohurler*):ab,ti,kw) NOT ((animal/exp OR animal*:de OR nonhuman/de) NOT ('human'/exp)) NOT [conference abstract]/lim

**Medline (Ovid)**

(exp Maxillofacial Abnormalities/ OR exp Mouth Diseases/ OR exp Tooth Diseases/ OR exp Mouth Abnormalities/ OR Micrognathism/ OR Prognathism/ OR Retrognathia/ OR Maxilla/ OR exp Surgery, Oral/ OR (maxill* OR macroglossia* OR macrostomia* OR microstomia* OR mouth* OR oral* OR lip* OR jaw* OR palate* OR tooth* OR teeth* OR dental* OR gingiva* OR tongue* OR enamel* OR temporomandibular-joint* OR stomatognathic-complex* OR caries* OR ((odontogenic* OR eruption* OR dentigerous*) ADJ3 (cyst*)) OR ((mandible*) ADJ3 hypoplasia*) OR micrognathia* OR prognathia* OR retrognathia*).ab,ti,kf.) AND (exp Mucopolysaccharidoses/ OR Mucolipidoses/ OR (mucolipidos* OR mucopolysaccharidos* OR MPS OR Scheie* OR (Hunter* ADJ3 (syndrome* OR disease*)) OR Hurler* OR Maroteaux-Lamy* OR Morquio* OR Sanfilippo OR Sly* OR natowicz* OR ((acetylglucosamin*) ADJ6 (phosphotransferas*) ADJ6 (syndrome* OR deficien*)) OR i-cell-disease* OR Leroy-disease* OR glcnac*-ptase*-deficien* OR GNPTAB OR GNPTG OR pseudohurler*).ab,ti,kf.) NOT (exp animals/ NOT humans/) NOT (news OR congres* OR abstract* OR book* OR chapter* OR dissertation abstract*).pt.

**Web of Science**

TS=(((maxill* OR macroglossia* OR macrostomia* OR microstomia* OR mouth* OR oral* OR lip* OR jaw* OR palate* OR tooth* OR teeth* OR dental* OR gingiva* OR tongue* OR enamel* OR temporomandibular-joint* OR stomatognathic-complex* OR caries* OR ((odontogenic* OR eruption* OR dentigerous*) NEAR/2 (cyst*)) OR ((mandible*) NEAR/2 hypoplasia*) OR micrognathia* OR prognathia* OR retrognathia*)) AND ((mucolipidos* OR mucopolysaccharidos* OR MPS OR Scheie* OR (Hunter* NEAR/2 (syndrome* OR disease*)) OR Hurler* OR Maroteaux-Lamy* OR Morquio* OR Sanfilippo OR Sly* OR natowicz* OR ((acetylglucosamin*) NEAR/5 (phosphotransferas*) NEAR/5 (syndrome* OR deficien*)) OR i-cell-disease* OR Leroy-disease* OR glcnac*-ptase*-deficien* OR GNPTAB OR GNPTG OR pseudohurler*)) NOT ((animal* OR rat OR rats OR mouse OR mice OR murine OR dog OR dogs OR canine OR cat OR cats OR feline OR rabbit OR cow OR cows OR bovine OR rodent* OR sheep OR ovine OR pig OR swine OR porcine OR veterinar* OR chick* OR zebrafish* OR baboon* OR nonhuman* OR primate* OR cattle* OR goose OR geese OR duck OR macaque* OR avian* OR bird* OR fish*) NOT (human* OR patient* OR women OR woman OR men OR man))) AND DT=(Article OR Review)

**Cochrane Central Registry of Trials**

((maxill* OR macroglossia* OR macrostomia* OR microstomia* OR mouth* OR oral* OR lip* OR jaw* OR palate* OR tooth* OR teeth* OR dental* OR gingiva* OR tongue* OR enamel* OR temporomandibular-joint* OR stomatognathic-complex* OR caries* OR ((odontogenic* OR eruption* OR dentigerous*) NEAR/3 (cyst*)) OR ((mandible*) NEAR/3 hypoplasia*) OR micrognathia* OR prognathia* OR retrognathia*):ab,ti,kw) AND ((mucolipidos* OR mucopolysaccharidos* OR MPS OR Scheie* OR (Hunter* NEAR/3 (syndrome* OR disease*)) OR Hurler* OR Maroteaux-Lamy* OR Morquio* OR Sanfilippo OR Sly* OR natowicz* OR ((acetylglucosamin*) NEAR/6 (phosphotransferas*) NEAR/6 (syndrome* OR deficien*)) OR i-cell-disease* OR Leroy-disease* OR (glcnac* NEXT ptase* NEXT deficien*) OR GNPTAB OR GNPTG OR pseudohurler*):ab,ti,kw)

**Google Scholar**

Maxilla|maxillofacial|mouth|oral|lip|jaw|palate|tooth|teeth|dental|gingiva|tongue|enamel mucolipidosis|mucopolysaccharidosis|Scheie|“Hunter syndrome|disease”|Hurler|Maroteaux-Lamy|Morquio|Sanfilippo|Sly|natowicz

**S2**

**Supplemental 2** List of all included references

1. Ak MM, George G, Al-Bahlani SS, Al Nabhani MZ. Difficult intubation management in a child with I-cell disease. Saudi J Anaesth. 2010;4(2):105-7.

2. Alpöz AR, Çoker M, Çelen E, et al. The oral manifestations of Maroteaux-Lamy syndrome (mucopolysaccharidosis VI): A case report. Oral Surg Oral Med Oral Pathol Oral Radiol Endod. 2006;101(5):632-7.

3. Antunes LA, Nogueira AP, Castro GF, Ribeiro MG, de Souza IP. Dental findings and oral health status in patients with mucopolysaccharidosis: a case series. Acta Odontol Scand. 2013;71(1):157-67.

4. Aviad I, Stein H, Zilberman Y. Roentgen findings of pseudo Hurler polydystrophy in the adult, with a note on cephalometric changes. Amer J Roentgenol. 1974;122(1):56-66.

5. Ballıkaya E, Eymirli PS, Yıldız Y, et al. Oral health status in patients with mucopolysaccharidoses. Turk J Pediatr. 2018;60(4):400-6.

6. Barker D, Welbury RR. Dental findings in Morquio syndrome (mucopolysaccharidoses type IVa). ASDC J Dent Child. 2000;67(6):431-3, 07.

7. Cavalcante WC, Santos LCS, dos Santos JN, et al. Oral findings in patients with mucolipidosis type III. Braz Dent J. 2012;23(4):461-6.

8. Cavaleiro RMDS, Pinheiro MDGR, Pinheiro LR, et al. Dentomaxillofacial manifestations of mucopolysaccharidosis VI: Clinical and imaging findings from two cases, with an emphasis on the temporomandibular joint. Oral Surg Oral Med Oral Pathol Oral Radiol. 2013;116(2):e141-e8.

9. de Almeida-Barros RQ, de Medeiros PFV, de Almeida Azevedo MQ, et al. Evaluation of oral manifestations of patients with mucopolysaccharidosis IV and VI: clinical and imaging study. Clin Oral Investig. 2018;22(1):201-8.

10. de Almeida-Barros RQ, Oka SC, Pordeus AC, et al. Oral and systemic manifestations of mucopolysaccharidosis type VI: a report of seven cases. Quintessence Int. 2012;43(3):e32-8.

11. de Oliveira Torres R, Pintor AVB, Guedes FR, et al. Three-dimensional dental and craniofacial manifestations in patients with late diagnosis of mucopolysaccharidosis type II: report of 2 cases. 2018.

12. Defraia E, Marinelli A, Antonini A, Giuntini V. Abnormal mandibular growth after craniovertebral surgery in Morquio syndrome type A. Angle Orthod. 2005;75(3):461-4.

13. Drążewski D, Grzymisławska M, Korybalska K, et al. Oral health status of patients with lysosomal storage diseases in Poland. Int J Environ Res Public Health. 2017;14(3).

14. Ferreira Dos Reis M, Pinheiro LR, Pinheiro MDGR, et al. Mucopolysaccharidosis VI and effects on growth of the apical bases: a case report. Spec Care Dentist. 2018;38(3):176-84.

15. Galili D, Yatziv S, Russell A. Massive gingival hyperplasia preceding dental eruption in I-cell disease. Oral Surg Oral Med Oral Pathol. 1974;37(4):533-9.

16. Gardner DG. The dental manifestations of the Morquio syndrome (mucopolysaccharidosis type IV). A diagnostic aid. Am J Dis Child. 1975;129(12):1445-8.

17. Gomez-Gonzalez A, Rosales-Berber MA, De Avila-Rojas P, Pozos-Guillen A, Garrocho-Rangel A. Pediatric Dental Management of an Uncommon Case of Mucopolysaccharidosis Type IV A (Morquio A Syndrome): A Case Report of a Three-Year Follow-Up. Case Reports in Dentistry. 2020;2020.

18. Gönüldaş B, Yilmaz T, Sivri HS, et al. Mucopolysaccharidosis: Otolaryngologic findings, obstructive sleep apnea and accumulation of glucosaminoglycans in lymphatic tissue of the upper airway. Int J Pediatr Otorhinolaryngol. 2014;78(6):944-9.

19. Guimarães MC, de Farias SM, Costa AM, de Amorim RF. Maroteaux-Lamy syndrome: orofacial features after treatment by bone marrow transplant. Oral Health Prev Dent. 2010;8(2):139-42.

20. Guven G, Cehreli ZC, Altun C, et al. Mucopolysaccharidosis type I (Hurler syndrome): oral and radiographic findings and ultrastructural/chemical features of enamel and dentin. Oral Surg Oral Med Oral Pathol Oral Radiol Endod. 2008;105(1):72-8.

21. Hingston EJ, Hunter ML, Hunter B, Drage N. Hurler's syndrome: Dental findings in a case treated with bone marrow transplantation in infancy. Int J Paediatr Dent. 2006;16(3):207-12.

22. Hopkins R, Watson JA, Jones JH, Walker M. Two cases of Hunter's syndrome—The anaesthetic and operative difficulties in oral surgery. British Journal of Oral Surgery. 1972.

23. James A, Hendriksz CJ, Addison O. The oral health needs of children, adolescents and young adults affected by a mucopolysaccharide disorder. JIMD rep. 2012;2:51-8.

24. Kantaputra PN, Kayserili H, Güven Y, et al. Oral manifestations of 17 patients affected with mucopolysaccharidosis type VI. J Inherit Metab Dis. 2014;37(2):263-8.

25. Kantaputra PN, Smith LJ, Casal ML, et al. Oral manifestations in patients and dogs with mucopolysaccharidosis Type VII. Am J Med Genet Part A. 2019;179(3):486-93.

26. Karir A, Geraghty M, Vassilyadi M, Doja A. Hemifacial spasm in mucopolysaccharidosis type VI (Maroteaux–lamy syndrome). Tremor Other Hyperkinetic Movements. 2018;8.

27. Kayserili H, Kantaputra PN. Multiple supernumerary molars, anterior openbite, and large ear lobules in mucopolysaccharidosis type VI patient. Am J Med Genet Part A. 2012;158 A(7):1798-800.

28. Keith O, Scully C, Weidmann GM. Orofacial features of Scheie (Hurler-Scheie) syndrome (α-L-iduronidase deficiency). Oral Surg Oral Med Oral Pathol. 1990;70(1):70-4.

29. Khadembaschi D, Arvier J, Beech N, Dolan D, McGill J. Successful management of severe coronoid process hyperplasia in a patient with mucopolysaccharidosis VI: A case report. J Surg Case Rep. 2020;2020(1).

30. Khalifa H, Grubisa HS, Lee L, Lam EW. Enlarged follicles and temporomandibular joint abnormalities in mucolipidosis Type III. Dentomaxillofac Radiol. 2013;42(4):22822014.

31. Kreidstein A, Boorin MR, Crespi P, Lebowitz P. Delayed awakening from general anaesthesia in a patient with Hunter syndrome. Canadian J Anaesth. 1994.

32. Kuratani T, Miyawaki S, Murakami T, Takano-Yamamoto T. Early orthodontic treatment and long-term observation in a patient with Morquio syndrome. Angle Orthod. 2005;75(5):881-7.

33. Lee W, O'Donnell D. Severe gingival hyperplasia in a child with I-cell disease. Int J Paediatr Dent. 2003;13(1):41-5.

34. Levin LS, Jorgenson RJ, Salinas CF. Oral findings in the Morquio syndrome (mucopolysaccharidosis IV). Oral Surg Oral Med Oral Pathol. 1975;39(3):390-5.

35. Liu KL. The oral signs of Hurler-Hunter syndrome: report of four cases. ASDC J Dent Child. 1980;47(2):122-7.

36. Lustmann J, Bimstein E, Yatziv S. Dentigerous cysts and radiolucent lesions of the jaw associated with Hunter's syndrome. J Oral Surg. 1975;33(9):679-85.

37. MacLeod SPR, Macintyre DR. Bilateral hypoplasia of mandibular condyles in Hurler's syndrome. Oral Surg Oral Med Oral Pathol. 1993;75(5):659-60.

38. McGovern E, Owens L, Nunn J, et al. Oral features and dental health in Hurler Syndrome following hematopoietic stem cell transplantation. Int J Paediatr Dent. 2010;20(5):322-9.

39. Melo MD, Obeid G. Radiolucent lesions of the maxillofacial complex in a patient with mucolipidosis type II (MLSII): case report. Oral Surg Oral Med Oral Pathol Oral Radiol Endod. 2007;104(4):e30-e3.

40. Mohammadi F, Tavakoli I. The oral and maxillofacial manifestation and surgical consideration in Maroteaux Lamy syndrome (mucopolysaccharidosis VI): A case report. Bioscience Biotechnology Research Communications. 2017;10(2):182-6.

41. Nakamura T, Miwa K, Kanda S, et al. Rosette formation of impacted molar teeth in mucopolysaccharidoses and related disorders. Dentomaxillofac Radiol. 1992;21(1):45-9.

42. Nayak DR, Balakrishnan R, Adolph S. Endoscopic adenoidectomy in a case of Scheie syndrome (MPS IS). Int J Pediatr Otorhinolaryngol. 1998.

43. Nelson J, Kinirons M. Clinical findings in 12 patients with MPS IV A (Morquio's disease). Further evidence for heterogeneity. Part II: Dental findings. Clin Genet. 1988;33(2):121-5.

44. Ribeiro EM, Fonteles CSR, Freitas AB, et al. A clinical multicenter study of orofacial features in 26 Brazilian patients with different types of mucopolysaccharidosis. Cleft Palate-Craniofac J. 2015;52(3):352-8.

45. Roberts MW, Barton NW, Constantopoulos G. Occurrence of multiple dentigerous cysts in a patient with the Maroteaux-Lamy syndrome (mucopolysaccharidosis, type VI). Oral Surg Oral Med Oral Pathol. 1984;58(2):169-75.

46. Rølling I, Clausen N, Nyvad B, Sindet-Pedersen S. Dental findings in three siblings with Morquio's syndrome. Int J Paediatr Dent. 1999;9(3):219-24.

47. Sabry S, Moheb D, Shahawy OE. Case report: Dentigerous cyst marsupialization for a child with hunter’s syndrome [version 1; peer review: 1 approved, 1 approved with reservations, 1 not approved]. F1000 Res. 2018;7.

48. Sela M, Eidelman E, Yatziv S. Oral manifestations of Morquio's syndrome. Oral Surg Oral Med Oral Pathol. 1975;39(4):583-9.

49. Shah AA, Hakim TA, Farooq S, et al. Multiple Dentigerous Cysts as a Rare Presentation of Maroteaux-Lamy Syndrome. Ann maxillofac surg. 2017;7(2):282-6.

50. Smith KS, Hallett KB, Hall RK, Wardrop RW, Firth N. Mucopolysaccharidosis: MPS VI and associated delayed tooth eruption. Int J Oral Maxillofac Surg. 1995;24(2):176-80.

51. Taylor NG, Shuff RY. I-Cell disease: an unusual cause of gingival enlargement. Br Dent J. 1994;176(3):106-8.

52. Thakur AR, Naikmasur VG, Sattur A. Hurler syndrome: orofacial, dental, and skeletal findings of a case. Skelet Radiol. 2015;44(4):579-86.

53. Torres RO, Pintor AVB, Guedes FR, et al. Oral and craniofacial manifestations in a Hunter syndrome patient with hematopoietic stem cell transplantation: A case report. Spec Care Dentist. 2018;38(1):51-4.

54. Wadenya RO, Stout AM, Gupta A, Monge J. Hurler syndrome: A case report of a 5-year follow-up of dental findings after bone marrow transplantation. Spec Care Dentist. 2010;30(1):14-7.

55. Webman MS, Hirsch SA, Webman H, Stanley HR. Obliterated pulp cavities in the Sanfilippo syndrome (mucopolysaccharidosis III). Oral Surg Oral Med Oral Pathol. 1977;43(5):734-8.

56. Yoon JH, Lee HI, Jang JH, et al. Oral manifestation and root canal therapy of the patient with mucopolysaccharidosis. Restor dent endod. 2019;44(2):e14.

57. Zolkipli Z, Noimark L, Cleary MA, Owens C, Vellodi A. Temporomandibular joint destruction in mucolipidosis type III necessitating gastrostomy insertion. Eur J Pediatr. 2005;164(12):772-4.

**S3**

**Supplemental Table 1** Number of patients reported with abnormal facial characteristics

|  | MPS I (n = 57) | MPS II (n = 45) | MPS III (n = 55) | MPS IV (n = 83) | MPS VI (n = 85) | MPS VII (n = 4) | ML II (n = 6) | ML III (n = 5) |
| --- | --- | --- | --- | --- | --- | --- | --- | --- |
| **Forehead** |  |  |  |  |  |  |  |  |
| Prominent | 2 | - | - | - | 4 | 1 | 1 | - |
| Narrow | - | - | - | - | - | - | 2 | - |
| **Eyes** |  |  |  |  |  |  |  |  |
| Hypertelorism | 3 | - | 1 | - | 8 | 2 | - | 2 |
| Exophthalmos | - | - | - | - | 3 | - | 1 | 1 |
| Periorbital edema | - | 1 | - | - | - | - | - | 1 |
| Oblique palpebral fissures | 1 | - | - | - | - | - | - | 2 |
| Puffy eyelids | 1 | - | - | - | - | - | 1 | - |
| Epicanthic folds | - | - | - | - | - | 1 | - | - |
| Deep nasojugal fold | - | - | - | - | 1 | - | - | - |
| Bushy eyebrows / eyelashes | 1 | - | - | - | 6 | - | - | - |
| Prominent supra-orbital ridges | 1 | 1 | - | - | - | - | - | - |
| **Ears** |  |  |  |  |  |  |  |  |
| Low | - | - | - | - | - | 1 | 1 | 2 |
| Enlarged / thickened earlobes | 1 | - | - | - | 3 | - | - | 1 |
| **Nose** |  |  |  |  |  |  |  |  |
| Flattened / depressed nasal bridge | 6 | 1 | 1 | 5 | 7 | 1 | 3 | - |
| Short nasal bridge | - | - | - | - | 2 | - | - | - |
| Broad nasal bridge | - | - | - | - | 1 | - | 1 | 1 |
| Broad nasal tip | 2 | 1 | - | - | 3 | - | - | 2 |
| Upturned nasal tip | 3 | - | - | 4 | - | - | - | 1 |
| Wide nostrils / flared nose | 2 | 1 | - | 4 | - | - | - | - |
| Saddle nose | - | - | - | - | 1 | - | - | 1 |
| Closed nasolabial angle | - | - | - | 1 | - | - | - | - |
| **Prominent cheeks** | - | - | - | - | 4 | - | - | 2 |
| **Philtrum** |  |  |  |  |  |  |  |  |
| Elongated | 1 | - | - | - | - | 1 | 1 | - |
| Flattened | 1 | - | - | - | - | - | - | - |
| **Lips** |  |  |  |  |  |  |  |  |
| Thickened | 6 | 12 | 2 | 5 | 10 | - | - | - |
| Retracted | - | - | - | - | 2 | - | - | - |
| Protruded | - | - | - | 1 | - | - | 1 | - |
| Broad | 2 | - | - | 1 | - | - | - | - |
| Long upper lip | 2 | - | - | - | - | - | - | - |
| Thin upper lip | - | - | - | - | - | 1 | - | - |
| Incompetent | 2 | 4 | 1 | 1 | 3 | - | 1 | - |
| **Apparent large mouth** | 2 | - | - | 4 | - | - | - | 1 |
| **Face** |  |  |  |  |  |  |  |  |
| Loss of vertical face height | - | - | - | 3 | - | - | - | - |
| Long face | - | - | - | - | 2 | - | - | 1 |
| Hypoplastic midface | 4 | 7 | 3 | 5 | 6 | 1 | - | - |
| Convex profile | 4 | 8 | 3 | 7 | 7 | - | - | - |
| Prominent lower third | 3 | 5 | 2 | 8 | 3 | - | - | - |
| **Hypertrichosis** | - | - | - | - | 4 | 1 | - | - |
